# Sleep Architecture and Daytime Sleepiness in Patients with Erectile Dysfunction

**DOI:** 10.3390/life13071541

**Published:** 2023-07-11

**Authors:** Helena Martynowicz, Rafal Poreba, Tomasz Wieczorek, Zygmunt Domagala, Robert Skomro, Anna Wojakowska, Sylwia Winiewska, Piotr Macek, Grzegorz Mazur, Paweł Gac

**Affiliations:** 1 Department of Internal Medicine, Occupational Diseases, Hypertension and Clinical Oncology, Wroclaw Medical University, Borowska 213, 50-556 Wroclaw, Poland; 2 Department of Psychiatry, Wroclaw Medical University, Pasteura 10, 50-367 Wroclaw, Poland; 3 Department of Anatomy, Wroclaw Medical University, Chałubińskiego 6a, 50-368 Wroclaw, Poland; 4 Division of Respiratory Critical Care and Sleep Medicine, University of Saskatchewan, Saskatoon, SK S7N 5E5, Canada; 5 Department of Experimental Dentistry, Wroclaw Medical University, Krakowska 26, 50-425 Wroclaw, Poland; 6 Division of Environmental Health and Occupational Medicine, Department of Population Health, Wroclaw Medical University, Mikulicza-Radeckiego 7, 50-368 Wroclaw, Poland

**Keywords:** hypertension, excessive daytime sleepiness, sleep apnea, sexual dysfunction

## Abstract

Obstructive sleep apnea is considered a risk factor for erectile dysfunction. The aim of this study was to determine sleep architecture and assess daytime sleepiness in patients with erectile dysfunction. The study group included 280 patients. The 107 enrolled patients had reported erectile dysfunction. The control group consisted of 173 patients who had no history of erectile dysfunction. The Epworth sleepiness scale (ESS) was used to measure the subjects’ level of daytime sleepiness. All patients underwent a standardized overnight, single-night polysomnography in sleep laboratory. In the erectile dysfunction group, we observed increased ESS total score and N1 sleep phase duration. Mean and minimal oxygen saturation and mean oxygen desaturation were decreased in comparison to the control group. In summary, subjects with erectile dysfunction have altered sleep architecture, oxygen saturation parameters and increased daytime sleepiness.

## 1. Introduction

Erectile dysfunction (ED) is defined as the consistent inability to obtain and/or maintain a penile erection during sexual activity [1]. ED affects millions of middle-aged to elderly men worldwide [2]. There are many causes of ED including diabetes, ischemic heart disease, medications (e.g., thiazides, b-blockers, spironolactone, and antidepressants), neurogenic disorders, atherosclerosis, tobacco use, hyperlipidemia, hypogonadism, lower urinary tract symptoms, metabolic syndrome, and depression [3,4]. The prevalence of ED increases with age, particularly after the age of 60 years [4,5,6]. There also data suggesting that obstructive sleep apnea (OSA) may have an independent association with sexual dysfunction and impotence [7,8]. OSA is a common sleep disorder characterized by the collapse of the upper airway leading to the cessation of airflow, intermittent arterial oxygen desaturation and arousals during sleep. Recent evidence showed that one in five adults suffer from at least a mild degree of OSA, 936 million adults aged 30–69 years have mild-to-severe OSA and 425 million (399–450) adults aged 30–69 years have moderate-to-severe obstructive sleep apnea globally [9,10]. Thus, this is one of the most common sleep disorders. Male sex and obesity are known risk factors for sleep apnea [11]. Several studies confirmed the increased prevalence of ED in patients with OSA [12,13].

Overnight polysomnography (PSG) is a gold standard in diagnosis of OSA [14]. Due to limited accessibility of PSG and its high expenditure, alternative tools for screening purposes have been developed including the Epworth sleepiness scale, Berlin Questionnaire or STOB-BANG Questionnaire. One of the most widely used is the Epworth sleepiness scale (ESS), which measures general level of sleepiness. The scale is self-administered: patients estimate their probability of falling asleep during different situations. The tool has been used in normal subjects [15], as well as in those with OSA [16], narcolepsy [17], stroke [18], coronary artery disease [19], heart failure [20], epilepsy [21], Parkinson’s disease [22], hemodialysis [23], diabetes [24], rheumatoid arthritis [25] and obesity [24]. However, the data concerning daytime sleepiness in erectile dysfunctions are limited. 

The aims of this study were as follows: 1. to determine sleep architecture in ED patients; and to 2. assess sleepiness scores using the Epworth sleepiness scale (ESS) in ED patients.

## 2. Materials and Methods

**Figure 1:**
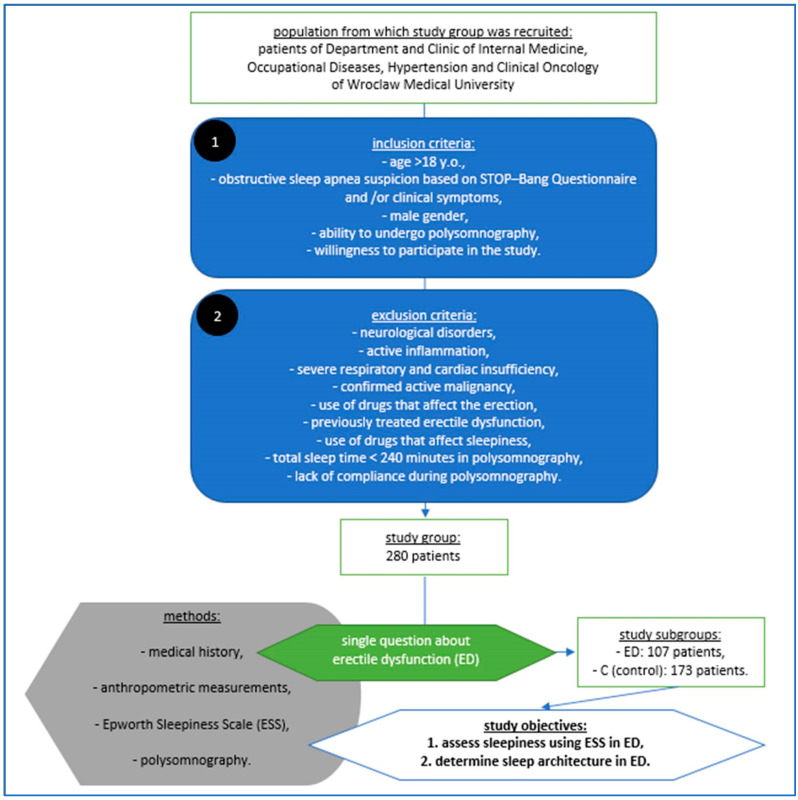
Material, methods and objectives of the study according to its protocol.

A summary of the study protocol is shown in Figure 1. 

The study group included 280 male patients of Department and Clinic of Internal Medicine, Occupational Diseases, Hypertension and Clinical Oncology hospitalized for the assessment of possible obstructive sleep apnea. The inclusion criteria obtain age > 18 years old, obstructive sleep apnea suspicion based on STOP–Bang Questionnaire and/or clinical symptoms, male gender, ability to undergo polysomnography and willingness to participate in the study, while the exclusion criteria included the presence of neurological disorders, active inflammation, severe respiratory and cardiac insufficiency, confirmed active malignancy, the use of drugs that affect the erection, previously treated erectile dysfunction, the use of drugs that affect sleepiness, in polysomnography total sleep time < 240 min and a lack of compliance during the study. The 107 enrolled patients had reported erectile dysfunction (ED). The control group (C) consisted of 173 patients who had no history of erectile dysfunction. ED was assessed via a single question during a clinical interview [26,27]. The Epworth sleepiness scale (ESS) was used to measure the subjects’ level of daytime sleepiness. The ESS was developed by Murray Johns at Epworth Hospital in Australia and was first reported in 1991. In the Epworth scale, the patients rate dozing in eight different situations. The minimum score of 0 indicates “would never doze”, while a maximum score of 3 indicates “high chance of dozing”. The total score can range from a minimum of 0 to a maximum of 24. Scores ≥ 10 on the ESS were indicative of excessive daytime sleepiness [15,16].

Height and weight were recorded using a nursing calibrated scale. The body mass index (BMI, calculated as weight in kilogram divided by square of height in meter) was calculated. Besides ESS, a questionnaire on OSA symptoms, OSA comorbidities, smoking status was performed. In the study group, 37.50% were smokers (*n* = 105), 78.21% hypertensives (*n* = 219), 12.85% patients had coronary heart disease (*n* = 36), 6.7% patients had a history of myocardial infarction (*n* = 19), and 8.2% were assessed after a stroke (*n* = 23). The mean score of the Epworth scale was 9.33 ± 5.40. The mean PSG parameters of the entire study group are presented in Table 1.

All patients underwent a standardized overnight, single-night polysomnography in a sleep laboratory. We used the NOXA1 (NOX Medical) PSG system. Polysomnograms were assessed in 30 s epochs according to the AASM (American Academy of Sleep Medicine) standard criteria for sleep scoring. PSG outcome variables included sleep latency, total sleep time (TST) and sleep efficiency (%), the ratio of N1, N2, N3 and the stage of REM. Abnormal respiratory events were scored from the pressure airflow signal evaluated according to the standard criteria of the American Academy of Sleep Medicine Task Force [28]. Apneas were defined as the absence of airflow for ≥10 s. Hypopnea was defined as a reduction in the amplitude of breathing by ≥30% for ≥10 s with a decline ≥3% in blood oxygen saturation or an arousal. A NONIN WristOx2 3150 pulse oximeter (Nonin Medical Inc., Plymouth, MN, USA), coupled with the PSG system, was used to record the oxygen saturation level. To analyze the full polysomnography recording, the Noxturnal software (Nox Medical, Reykjavík, Iceland) was used. A certified, qualified physician (H.M.) from the sleep laboratory scored and manually analyzed the data in accordance with the AASM guidelines.

Statistical analysis was conducted using the statistical software Statistica 12 PL, Statistica, Tulsa, US. For quantitative variables, arithmetic means and standard deviations were calculated for the estimated parameters in the studied groups. The distribution of the variables was tested using the Shapiro–Wilk test. In cases of quantitative variables manifesting the normal distribution in further statistical analysis, the *t* test for unlinked variables was used. In cases of variables manifesting distribution distinct from the normal one, the nonparametric equivalent of the *t* test, i.e., the Mann–Whitney U test was used. In order to detect relationships between the studied variables, univariate regression analysis was performed. The results at the level of *p* < 0.05 were accepted as statistically significant.

This study was approved by the Ethical Committee of Wroclaw Medical University (ID KB-227/2015) and was conducted in accordance with the Declaration of Helsinki. All patients signed an informed consent form for this study.

## 3. Results

The mean BMI was higher in the ED group compared to the that of the control. The mean age, BMI, height, body mass and smoking status are shown in Table 2.

In the ED group, statistically significantly AHI (apnea/hypopnea index) < 5 (AHI excluding OSA) was observed less frequently than in the control group. The number of subjects according to AHI in patients with erectile dysfunction (ED) and control (C) subjects is shown in Table 3.

In the erectile dysfunction group (ED), we observed a higher ESS total score and lower mean and minimal oxygen saturation levels compared to those of the control group. The mean oxygen desaturation levels were lower in the ED group in comparison with the control group. The N1 sleep phase was increased in comparison with the control group. The polysomnographic parameters and ESS (Epworth sleepiness scale) score in patients with erectile dysfunction (ED) and control (C) subjects are presented in Table 4.

Then, we performed an additional analysis considering the occurrence of hypertension. Hypertension is an important risk factor for erectile dysfunction and may also affect the sleep architecture and the level of sleepiness. Therefore, it might affect the results of the study. We divided all participants into two subgroups (normotensive and hypertensives). In univariate regression analysis, in the hypertensive group, we observed a relationship between the presence of erectile dysfunction and mean saturation (r = −0.20; *p* < 0.05), minimal saturation (r = −0.14; *p* < 0.05), and N1 (%TST) (r = 0.31, *p* < 0.05). In the normotensive group, we observed a relationship between ED and AHI (apnea/hypopnea index) (r = 0.43, *p* < 0.05), ED and ODI (r = 0.45, *p* < 0.05) and ED and TST (r = −0.82, *p* < 0.05). In this group, we also observed a relationship between ED and mean saturation (r = −0.44, *p* < 0.05) and minimal saturation (r = −0.45, *p* < 0.05). Interestingly, in the normotensive group, we observed a relationship between ED and N2 (%TST) (r = −0.57, *p* < 0.05), N3 (%TST) (r = 0.62, *p* < 0.05) and REM (rapid eye movement) sleep (%TST) (r = −0.61, *p* < 0.05). The result of the univariate regression analyses in the hypertensive and normotensive groups are summarized in Table 5.

## 4. Discussion

Obstructive sleep apnea affects both the patient and the sexual partner [29,30,31]. In a cross-sectional study of 401 men referred to a sleep lab for suspected OSA, 92% were diagnosed with OSA, and ED was present in 69% of those with OSA and 34% without OSA [8]. The evidence for the association between OSA and ED remains inconclusive, with some studies suggesting no association, or an association being shown for severe OSA only [32,33]. The pathogenesis of ED in OSA is complex. The factors implicated include increased sympathetic activity, oxidative stress, endothelial dysfunction a reduction in nitric oxide formation [34,35]. Sleep fragmentation and a lack of rapid eye movement (REM) periods related to OSA may be involved in ED pathogenesis because physiological erections appear to help maintain erectile function through cavernous tissue oxygenation during the REM sleep period [36,37]. ED is also related to changes in the hormonal axis caused by sleep pattern changes associated with OSA [38].

The main result of this study is an increased ESS total score in patients with erectile dysfunction compared to those of the control group, although the AHI and ODI (oxygen desaturation index) were similar in both groups. It is worth noting that this is a novel observation. In the ED group, the mean saturation, minimal saturation and mean desaturation levels were decreased compared to those of control group. Interestingly, we also observed increased N1 sleep duration (%TST) in the ED group. N1 is the lighter stage of NREM sleep, which usually occurs at the beginning of sleep and often alternates with brief arousal episodes. N1 is the period of transition from unsynchronized beta and gamma brain waves to more synchronized but slower alpha waves, and then to theta waves with slow rolling eye movement. This stage comprises only about 5% of the total sleep time. In our study, N1 comprises as much as 17.3% of TST in patients with erectile dysfunction. This phenomenon may be a consequence of increased activity of the sympathetic nervous system, which is common in OSA due to repeated hypoxemia. Additionally, fatigue and a decrease in rapid eye movement (REM) sleep period may provoke the deterioration in the quality of erections [39,40]. These results agree with those of our study. We observed a negative relationship between ED and the REM (rapid eye movement) period (%TST); however, we did not observe statistical differences between the REM period in ED and controls.

In our study, the patients with ED had lower mean oxygen saturation, minimal saturation and mean desaturation levels compared to the control group. These results are in agreement with those of previous studies, which showed that recurrent apnea attacks in patients with OSA cause hypoxia reperfusion injury and oxidative stress, endothelial dysfunction and, consequently, ED [41]. Severe and moderate OSA was more prevalent in the ED group than in the control group, which may be a possible explanation for these observations.

OSA is a known risk factor for erectile dysfunction; however, the mechanism underlying ED in patients with OSA is complex and remains unclear. The data on EDS (excessive daytime sleepiness) and ED are limited and often contradictory. Surprisingly, it was demonstrated that ED subjects had significantly lower ESS and SaO_2_ [42]. Recently. Jeon also showed that ESS is inversely correlated with the International Index of Erectile Function (KIIEF-5) [43]. We have shown that patients with ED have increased total ESS scores compared to those of the control group, which is a novel observation. This is most likely the result of increased frequency of severe and moderate OSA in the ED group. However, we have not observed a statistically significant difference in AHI between the ED and control groups; thus, other mechanisms may be involved.

The most unexpected result of our study is the presence of a relationship between ED and N3 sleep (%TST) in normotensive patients; however, the duration of N3 was similar between the ED group and the control group. We did not observe this relationship in hypertensive patients, which might suggest different relationships between the structure of sleep and ED depending on the blood pressure levels. We have also observed a positive relationship between AHI, ODI (oxygen desaturation index), and erectile dysfunction, and a negative relationship between mean saturation, minimal saturation, TST and erectile dysfunction in the normotensive group. The relationship between AHI, ODI, TST and ED was not observed in hypertensive patients.

The present study confirms the strong role of hypoxia in erectile dysfunction. Firstly, we observed decreased mean oxygen saturation as well as minimal oxygen saturation levels in the erectile dysfunction group compared to those of the control group. Secondly, both in hypertensive and normotensive patients, mean and minimal oxygen saturation were related to erectile dysfunction. Chronic hypoxia may be observed in physiological conditions such as aging, as well as in many pathological conditions such as smoking and chronic obstructive pulmonary disease (COPD), heart and respiratory failure, obstructive sleep apnea, atherosclerosis, diabetes, and hypertension. It is worth noting that CPAP (continuous positive airway pressure), when used with testosterone replacement therapy, can increase total testosterone and cause an improvement in the indicators of altered nocturnal penile erection episodes [44]. In the present study’s regression analysis, the oxygen desaturation index was associated with ED only in the normotensive group. These results may indicate the role of oxygen level drops in normotensive but not in hypertensive patients. Recently, Feng demonstrated that minimum oxygen saturation and average oxygen saturation may predict the occurrence of ED in obstructive sleep apnea patients [45]. The possible mechanisms involved in hypoxia on erectile dysfunction mainly include excessive reactive-oxygen-species-mediated oxidative stress, hypoxia-inducible factors-1α mediated endothelial cell apoptosis and proliferation inhibition, endothelial dysfunction, reduced nitric oxide production and systematic inflammation [46].

Short sleep duration is considered a risk factor for erectile dysfunction. The sleep duration recommended by the AASM is between 7 and 9 h of sleep. A short sleep duration is defined as habitual sleep time of less than 6 h [47]. It was previously shown that short sleep is associated with a large group of disorders, such as hypertension, diabetes, major depressive disorder, and other morbidities [48,49]. Sleep duration is decreasing in modern societies, as large cohorts studies have shown [50,51,52]. As much as 29.1% of adults suffered from short sleep duration is United States [53]. The pathomechanisms of erectile dysfunction in short sleepers are complex and lead to hypothalamic–pituitary–adrenal axis overactivity, resulting in cortisol release as well as an autonomic nervous system imbalance, consequently resulting in catecholamines release and vasoconstriction. Moreover, short sleep duration may reduce the testosterone level and the frequency of REM sleep and sleep-related erections (SREs) [54]. SREs or nocturnal penile tumescence (NPT) can be measured using PSG. The majority of NPT occurrences, which are a physiological and spontaneous phenomenon, are related to REM sleep [55]. The erection initiates during the shift from NREM to REM sleep with full tumescence throughout REM; however, in the present study, we did not measure NPT. It is worth noting that short sleep duration is related to many negative health outcomes such as cardiovascular diseases, increased morbidity and mortality [56,57]. In the present study, we did not observe statistically significant differences in total sleep time between the patients with erectile dysfunction and the control group. Although sleep time was comparable, we noticed an increased stage N1 sleep duration. A high percentage of the stage N1 sleep is usually a result of frequent arousals caused by sleep disorders or environmental disturbances. Thus, in the present study, altered sleep structure, but not sleep duration, was related to erectile dysfunction.

Summarizing, we studied the sleep structure and level of sleepiness of a relatively large ED cohort using polysomnography, which is the gold standard for sleep assessment. We have confirmed many observations that have been described before, especially the relationship between OSA and ED; however, this study has some novel conclusions. We have also observed an increased sleepiness level in ED subjects in comparison to that of the control group. We have shown the importance of oxygen saturation parameters and altered sleep architecture in erectile dysfunction. We have also confirmed the relationship between ED and sleep parameters in normotensive but not in hypertensive patients. These results indicate a different mechanism of ED in hypertension; thus, further studies are needed to explain these observations.

The strengths of this study are its large population and use of the gold standard in sleep disorders diagnosis (avPSG). However, several limitations of the present study should be highlighted. Firstly, we did not use any scales for measuring erectile dysfunction, including the International Index of Erectile Dysfunction (IIED), which may be a major confounder. The cause, type and degree of severity of ED was not studied. We did not collect data on hypotensive therapy, which could affect erectile dysfunction. In addition, there was a small number of patients in the group without OSA compared to the OSA group.

## 5. Conclusions

Patients with erectile dysfunction are more likely to be affected by obstructive sleep apnea, altered sleep architecture and oxygen saturation parameters compared to the control group. The sleepiness measured using ESS in patients with erectile dysfunction is increased compared to that of the control group.

## Figures and Tables

**Table 1:** The PSG parameters of the entire study group.

PSG Parameter	Mean Value	SD
TST (min)	384.53	108.77
Sleep latency (min)	31.0	31.87
SE (%)	73.82	18.82
AHI (*n*/h)	21.83	20.63
Mean O_2_ Sat (%)	93.71	2.84
ODI (min/h)	20.44	21.17
Mean desaturation (%)	4.95	1.81
Min O_2_ Sat(O_2_)	81.16	8.80
SatO_2_ < 90% (%)	14.85	22.21
N1 (%TST)	11.73	14.14
N2 (%TST)	49.94	18.93
N3 (%TST)	19.56	17.73
REM (%TST)	19.21	10.18

PSG—polysomnography; TST—total sleep time; AHI—apnea/hypopnea index; ODI—oxygen desaturation index; mean SpO_2_—mean oxygen saturation; min SpO_2_—minimal oxygen saturation; REM—rapid eye movements.

**Table 2:** The height, body mass, BMI (body mass index), and smoking status of patients with erectile dysfunction (ED) and control (C) subjects.

	C (*n* = 173)	ED (*n* = 107)	*p*
Age (years)	48.49 ± 13.31	48.92 ± 10.30	ns
Height (cm)	177.13 ± 7.44	174.76 ± 6.84	<0.05
Body mass (kg)	95.83 ± 18.90	99.57 ± 17.49	ns
BMI (kg/m^2^)	30.50 ± 5.46	32.52 ± 4.73	<0.01
Smoking (years)	20.45 ± 11.70	23.55 ± 12.61	ns

**Table 3:** The number of subjects according to AHI in patients with erectile dysfunction (ED) and control (C) subjects.

AHI	C (*n* = 173)	ED (*n* = 107)	*p*
<5 (*n* = 44)	32 (24)	12 (13)	<0.05
>5 and <15 (*n* = 66)	39 (30)	27 (30)	ns
>15 and <30 (*n* = 51)	27 (21)	24 (26)	ns
>30 (*n* = 61)	33 (25)	28 (31)	ns

AHI—apnea/hypopnea index.

**Table 4:** The polysomnographic parameters and ESS (Epworth sleepiness scale) score in patients with erectile dysfunction (ED) and control (C) subjects.

	C (*n* = 173)	ED (*n* = 107)	*p*
ESS score (points)	8.87 ± 5.33	10.07 ± 5.44	<0.05
TST (min)	399.92 ± 110.83	360.55 ± 102.35	ns
Sleep latency (min)	29.38 ± 31.26	33.75 ± 33.13	ns
AHI (/h)	20.41 ± 20.65	23.87 ± 20.54	ns
Mean SpO_2_(%)	94.19 ± 2.12	92.98 ± 3.57	<0.01
Min SpO_2_(%)	82.24 ± 2.11	79.56 ± 9.68	<0.05
ODI (/h)	18.85 ± 20.63	22.84 ± 21.87	ns
Mean oxygen desaturation (%)	4.67 ± 1.79	5.42 ± 1.78	<0.05
Mean HR (bpm)	60.72 ± 8.62	62.09 ± 9.51	ns
N1 (%TST)	8.35 ± 6.93	17.34 ± 20.22	<0.01
N2 (%TST)	52.32 ± 19.24	46.01 ± 18.00	ns
N3 (%TST)	19.75 ± 17.87	19.26 ± 17.78	ns
REM (%TST)	20.35 ± 10.06	17.39 ± 10.26	ns
Sleep efficiency (%)	76.30 ± 17.78	70.06 ± 19.69	ns

TST—total sleep time; AHI—apnea/hypopnea index; ODI—oxygen desaturation index; Mean SpO_2_—mean oxygen saturation; Min SpO_2_—minimal oxygen saturation; REM—rapid eye movements.

**Table 5:** The results of univariate regression analyzes between erectile dysfunction (ED) and polysomnographic parameters in hypertensive and normotensive groups.

Dependent Variable	Independent Variable	Hypertensive Group	Normotensive Group
Presence of erectile dysfunction	ESS score (points)	ns	ns
	TST (min)	ns	r = −0.82
	Sleep latency (min)	ns	ns
	AHI (/h)	ns	r = 0.43
	Mean SpO_2_ (%)	r = −0.20	r = −0.44
	Min SpO_2_ (%)	r = −0.14	r = −0.45
	ODI (/h)	ns	r = 0.45
	Mean oxygen desaturation (%)	ns	ns
	Mean HR (bpm)	ns	ns
	N1 (%TST)	r = 0.31	ns
	N2 (%TST)	ns	r = −0.57
	N3 (%TST)	ns	r = 0.63
	REM (%TST)	ns	r = −0.61
	Sleep efficiency (%)	ns	ns

TST—total sleep time; AHI—apnea/hypopnea index; ODI—oxygen desaturation index; Mean SpO_2_—mean oxygen saturation; Min SpO_2_—minimal oxygen saturation; REM—rapid eye movements.

## Data Availability

The data presented in this study are available upon request from the corresponding author. The data are not publicly available.
